# Phosphoinositide 3-kinase p85beta regulates invadopodium formation

**DOI:** 10.1242/bio.20148185

**Published:** 2014-09-12

**Authors:** Ariel E. Cariaga-Martínez, Isabel Cortés, Esther García, Vicente Pérez-García, María J. Pajares, Miguel A. Idoate, Javier Redondo-Muñóz, Inés M. Antón, Ana C. Carrera

**Affiliations:** 1 Department of Immunology and Oncology, Centro Nacional de Biotecnología (CNB-CSIC), Campus de Cantoblanco, Madrid E-28049, Spain; 2 Department of Molecular and Cell Biology, Centro Nacional de Biotecnología (CNB-CSIC), Campus de Cantoblanco, Madrid E-28049, Spain; 3 Biomarkers Laboratory, Division of Oncology, Center for Applied Biomedical Research (CIMA), University of Navarra, Pamplona E-31008, Spain; 4 Pathology Department, Hospital Clinic of Navarra, University of Navarra, Pamplona, E-31008, Spain

**Keywords:** p85β, invadopodium, invasion, cell adhesion, metastasis

## Abstract

The acquisition of invasiveness is characteristic of tumor progression. Numerous genetic changes are associated with metastasis, but the mechanism by which a cell becomes invasive remains unclear. Expression of p85β, a regulatory subunit of phosphoinositide-3-kinase, markedly increases in advanced carcinoma, but its mode of action is unknown. We postulated that p85β might facilitate cell invasion. We show that p85β localized at cell adhesions in complex with focal adhesion kinase and enhanced stability and maturation of cell adhesions. In addition, p85β induced development at cell adhesions of an F-actin core that extended several microns into the cell z-axis resembling the skeleton of invadopodia. p85β lead to F-actin polymerization at cell adhesions by recruiting active Cdc42/Rac at these structures. In accordance with p85β function in invadopodium-like formation, p85β levels increased in metastatic melanoma and p85β depletion reduced invadopodium formation and invasion. These results show that p85β enhances invasion by inducing cell adhesion development into invadopodia-like structures explaining the metastatic potential of tumors with increased p85β levels.

## INTRODUCTION

Cell invasion is a complex process that involves cell adhesion, polarization and formation of invasive structures (Morgan et al., 2007; Block et al., 2008). Whereas adhesion is mediated through structures such as focal contacts, focal adhesions and fibrillar adhesions, invasion involves formation of deep, specialized adhesion-like structures such as podosomes and invadopodia. The relationship between cell adhesions, podosomes and invadopodia remains unclear (Block et al., 2008; Murphy and Courtneidge, 2011; Linder et al., 2011).

Cell adhesions are distributed as foci on the ventral cell surface, concentrating integrin receptors and cytoskeleton contractile fibers; they mediate cell attachment to the extracellular matrix (ECM) and, can also degrade ECM in some tumor cells (Morgan et al., 2007; Wang and McNiven, 2012; Wolfenson et al., 2013). There are several types of cell adhesions; focal contacts are shell-shaped adhesion structures that form immediately behind the cell leading edge and are transformed into longer structures termed focal adhesions that provide firm adhesion via actomyosin stress fibers. These focal adhesions in turn develop into centrally located fibrillar adhesions, sites of fibronectin matrix deposition that mediate strong adhesion. Cell adhesions are highly dynamic structures containing proteins that include focal adhesion kinase (FAK), talin, paxillin and vinculin, some of which connect integrin receptors with the actomyosin fibers (Morgan et al., 2007; Block et al., 2008; Wolfenson et al., 2013).

The structures formed during invasion (invadopodia and podosomes) differ from focal adhesions in their capacity to protrude into (and degrade) the ECM. Podosomes are ring-like structures that contain vinculin and paxillin; they are found in normal cells able to remodel tissues as well as in Src-transformed cells. Invadopodia are similar, but are found in invasive cancer cells. Unlike focal adhesions, podosomes and invadopodia stably bind active forms of the GTPases Cdc42 and Rac, which mediate formation of the F-actin skeleton that maintains their protruding structure (Tsuboi, 2007; Block et al., 2008; Albiges-Rizo et al., 2009).

The class I_A_ phosphoinositide 3-kinase (PI3K) pathway is critical in the regulation of cell migration and is often hyperactivated in cancer cells (Jiménez et al., 2000; Brachmann et al., 2005; Hoshino et al., 2012; Courtney et al., 2010). Class I PI3K are lipid kinases that generate PtdIns(3,4)P_2_ and PtdIns(3,4,5)P_3_ after activation of Tyr kinase or G protein-coupled receptors (Fayard et al., 2010). These lipids increase transiently after growth factor receptor stimulation and promote cell division, survival and migration *via* downstream effectors such as protein kinase B and Rho GTPases (Welch, et al., 2003; Vanhaesebroeck and Waterfield, 1999; Sanz-Moreno et al., 2008; Fayard et al., 2010). PI3K are comprised of a p85 regulatory and a p110 catalytic subunit. Three genes encode PI3K regulatory subunits, *PIK3R1* (p85α), *PIK3R2* (p85β) and *PIK3R3* (p55γ), all of which bind to one of the catalytic subunits (Vanhaesebroeck and Waterfield, 1999). p85α and p85β are ubiquitous and mediate p110 stability and activation (Inukai, et al., 1997; Yu et al., 1998).

Expression of p85α is generally higher than that of p85β in normal cells, whereas p85β becomes predominant in high-grade mammary and colon carcinomas (Cortés et al., 2012). The p85β mode of action in tumor progression remains unknown; we tested whether p85β promotes cell invasion. We show that p85β localizes at cell adhesions in complex with FAK. p85β expression stabilized focal adhesions and mediated formation of cell adhesions that extend several microns into the z-axis and have an F-actin core, similar to that of invadopodia. p85β depletion reduced the depth and GTP-Cdc42/Rac levels of cell adhesions, suggesting that p85β functions by recruiting these active GTPases to cell adhesions. p85β overexpression was frequent in metastatic melanoma, and its depletion in an invasive melanoma cell line impaired invadopodium formation and invasion. The presented observations suggest that when tumors increase p85β expression, this results in p85β constitutive localization at cell adhesions (in complex with FAK), which, in the presence of growth factors, enables accumulation of GTP-Cdc42/Rac at cell adhesions and generation of a z-axis F-actin core, necessary for invadopodium formation.

## MATERIALS AND METHODS

### Cells, cell culture and transfection

Murine embryonic fibroblasts (MEF) were prepared as reported (García et al., 2006) from p85α^−/−^ and p85β^−/−^ mice (Fruman et al., 1999; Deane et al., 2004). Freshly isolated WT, p85α^−/−^ and p85β^−/−^ MEF were cultured and used within two weeks. NIH3T3 and BLM cells were maintained in Dulbecco's modified Eagle medium supplemented with 10% fetal bovine serum, 2 mM glutamine, 10 mM Hepes, 100 U/ml penicillin and 100 µg/ml streptomycin. Cells were transfected with Lipofectamine (Invitrogen).

### cDNA and siRNA

We used pSG5 empty vector, pSG5-p85α and pSG5-Myc-Cdc42 or pSG5-V12-Cdc42 (Jiménez et al., 2000); GFP-paxillin was donated by Dr. M Ginsberg (University of California-San Diego, CA) and pT7/T3-U19 encoding murine p85β was a kind gift of Dr. JWG Janssen (Inst fur Humangenetik, Universitäts Klinikum, Heidelberg, Germany) (Janssen et al., 1998). p85β was subcloned into pSG5 and a hemagglutinin (HA) epitope added in-frame in the N terminus. The p85β ATG codon was replaced with a proline residue and the HA-tag ATG codon was maintained (Quickchange mutagenesis kit; Stratagene); Δp85β was prepared from this plasmid by introducing an HpaI site in positions +1383 and +1507 from the ATG codon, the cDNA was restricted with HpaI, and the resulting fragment lacking residues 461–502 (in the p85β inter-SH2 domain) was ligated. Human control and p85β siRNA were from Dharmacon. siRNA for murine FAK (Ptk2; SR421142) was from Origene.

### Antibodies and reagents, Western blot, immunoprecipitation and pull-down assays

Primary antibodies for Western blot (WB) and immunofluorescence (IF) were: anti-pan-p85 PI3K, -human p85α and -PKB (Upstate Biotechnology), anti-HA (12CA5; Babco) and -β-actin (Sigma–Aldrich). Anti-p85β PI3K (rat 1C8, Cortés et al., 2012) and -HA (12CA5) Ab were used for immunoprecipitation (IP) and WB. Anti-*cis*-Golgi Ab was from R&D Systems; anti-FAK Ab was from BD Bioscience; anti-pTyr576/577 FAK was from Cell signaling. The K1123 anti-p85β antibody was obtained by immunizing rabbits with a KLH-conjugated C-terminal peptide (residues 711–722; CRAPGPGPPSAAR), and was tested in ELISA, WB and IP using recombinant bacterial protein (GST-fused N-terminal fragment of murine p85β [residues 1–305] or extracts of r-p85β- or r-p85α-expressing cells). In WB, K1123 specifically recognized p85β. Phalloidin-TRITC, -FITC (fluorescein isothiocyanate) and FAK inhibitor (PF-573228) were from Sigma. PDGF-BB was from eBioscience and fibronectin from R&D Systems.

Cells were lysed in RIPA lysis buffer for WB and in TX-100 lysis buffer for IP; both buffers contained protease/phosphatase inhibitors and protocols were as described (Marqués et al., 2008). Pull-down assays were as described (Jiménez et al., 2000); we used glutathione S-transferase (GST) fused to the Pak1 CRIB domain (CRIB^Pak1^) as bait for Rac, GST-NWASP (CRIB^NWASP^) for Cdc42, and GST-rhotekin-RBD for RhoA.

### Immunofluorescence, immunohistochemistry and confocal microscopy

For IF, cells were fixed in fresh 4% paraformaldehyde (PFA) in PBS (15 min), permeabilized in PBS with 1% BSA and 0.3% Triton X-100, and blocked using 1% BSA, 10% goat serum and 0.01% Triton X-100 in PBS (30 min). Cells were incubated with primary antibody (30 min, room temperature) and appropriate secondary antibody as described (Jiménez et al., 2000). DNA was stained with Hoechst 33258 or DAPI. We stained p85α with a specific anti-human p85α or a pan-p85 antibody that recognizes p85α with higher affinity than p85β (Alcázar et al., 2007). For p85β, we used K1123; we also used anti pan-p85 antibody to stain invadopodia, as p85α did not recognize adhesion or invadopodial structures. Cells were visualized using a 60× 1.3NA PLOIL objective on an inverted Olympus Fluoview 1000 microscope; other images were collected on a confocal Leica SP5 TCS system equipped with a Leica HCX PL Apo CS lambda blue 63×/1.4NA oil objective lens. We used PBS/glycerol mounting media; all conditions were viewed with the same settings.

We used a malignant melanoma and normal tissue array (UB Biomax) containing 128 cases of primary malignant melanoma (grades I to IV), as well as 64 metastatic melanomas in duplicate and 16 samples of normal skin tissue (5 µm sections). Sections were deparaffinized, hydrated and endogenous peroxidase activity was inhibited using 3% H_2_O_2_. Microwave antigen retrieval was carried out with citrate buffer (10 mmol/L, pH 6), twice for 10 min. Sections were incubated with anti-p85β (1:50; K1123; overnight, 4°C). After washing the slide with TRIS-buffered saline, reactivity was detected with the EnVision HRP System (DAB; Dako). Staining scores were established as described (Pajares et al., 2012).

### Live-cell microscopy by total internal reflection fluorescence microscopy (TIRFM)

BLM cells (2×10^5^/well) were seeded onto a glass-bottomed p35 dish (Mattek Corporation). Prior to imaging, cells were transfected with a specific p85β siRNA or a negative control sequence (SC) (Stealth RNAi, Life Technologies). After 24 h, cells were transfected with a pEGFP plasmid (Clontech) containing human GFP-paxillin (24 h). Live cells were imaged on a Leica AF 6000LX microscope equipped with a TIRF illuminator, 100× 1.46 NA HCX PL APO objective, in a temperature/CO_2_-controlled chamber. Images were taken every 2 min for at least 90 min, and the time series analyzed using ImageJ software (NIH).

### Adhesion, migration and invasion assays

Adhesion assays were as described (Sánchez-Aparicio et al., 1994). For migration assays (Jiménez et al., 2000; Parmo-Cabañas et al., 2004), 5 µm-pore size transwells (Costar, Cambridge, MA), were incubated in PBS containing FN or collagen (10 µg/ml) by overnight incubation at 4°C; transwells were washed in PBS and then the cells (15×10^3^ MEF or fibroblasts) were plated on the top; chemoattractants (indicated) were added to the bottom chamber and migration assay was incubated for 5 h (5% CO_2_, 37°C). Under these conditions, the cells are platted in a small amount of FN or collagen (that helps them to attach) but do not require FN or collagen degradation to cross to the bottom chamber and after 5 h cell counting in the bottom chamber was up to 50% of the platted cells. Invasion assays on gelatin (supplementary material Fig. S3) were performed as described (Bartolomé et al., 2006; Molina-Ortiz et al., 2009; Cortés et al., 2012). Briefly, prewarmed gelatin (Millipore) was diluted to 50% in PBS (V/V) and 45 µl were added per well (24well, 5 µm-pore size Transwells, BD Falcon) and incubated for ∼1 h at 37°C, until matrix solidified. The lower compartmnet was filled with DMEM, 0.5% BSA, 50 ng/ml PDGF and 50×10^3^ cells were plated on the top; the assay was incubated at 37°C, 5% CO_2_. Under these conditions, the cells require to degrade the matrix to cross to the bottom chamber and after 22 h, maximal cell counting in the bottom chamber was up to ∼5×10^3^ cells (10% of the cells invaded under optimal conditions).

Invasion was also evaluated by measuring fluorescent matrix degradation; fluorescent matrix-coated dishes were prepared as described (Bowden et al., 2001). Gelatin was labeled with rhodamine B isothiocyanate in a buffer containing 50 mM Na_2_B_4_O_7_ and 61 mM NaCl, pH 9.3. Unbound dyes were removed by extensive dialysis against PBS (4°C, 2 days). Coverslips were acid-washed and coated with 100 µl pre-warmed rhodamine-gelatin (2 mg/ml), followed by crosslinking with 0.5% glutaraldehyde in PBS (15 min). After extensive washing with PBS, coverslips were treated with 5 mg/ml NaBH_4_ (3 min), washed with PBS, sterilized with 70% ethanol (5 min) and incubated in serum-free medium (1 h) before use. BLM cells were cultured on gelatin-coated coverslips (6 h), fixed in 4% PFA and processed for IF. Coverslips were mounted in Fluoromount-G medium (Southern Biotech). Images were collected using a Leica SP5 TCS confocal system (as above).

Basement membrane from murine peritoneum was isolated as described (Hotary et al., 2006). Membranes were mounted on transwell inserts and sealed with a 50:50 wax:paraffin mixture. After stripping the overlying mesothelial cells from the membrane using 1 N ammonium hydroxide (30 min) and washing with PBS (3.8 mM NaH_2_PO_4_, 16.2 mM Na_2_HPO_4_, 150 mM NaCl), membranes were sterilised using 70% ethanol. We seeded 2×10^5^ cells in 100 µl growth medium on the mebranes; 500 µl of the same medium were added to the bottom chamber. After 2–3 days invasion, samples were fixed and processed for IF. Membranes and cells were stained for type IV collagen, and DAPI and visualized as above. Serial optical sections were captured along the BM at 2-µm intervals. Remaining type IV collagen in the membrane was measured in 8-bit Z-projections.

### Statistical analyses

To measure the depth of cell adhesions, we counted the number of sections (with at least a three-fold increase in phalloidin or paxillin signal over background) and multiplied this value by the step size between sections. Associations between tumor variables were assessed by Student's *t*-test (two-tailed) and ANOVA, calculated using Prism5V.5.0b software. Methods for TIRFM image quantitation have been described (Berginski et al., 2011); briefly, image stacks were thresholded to avoid spurious signals and several cell adhesion kinetic properties were measured over time. We include at least three cells from independent experiments for each condition studied. Data were analyzed using the Wilcoxon test for paired samples.

## RESULTS

### Distinct intracellular localization of p85β and p85α

Cell invasion is a process that coordinates cell adhesion with formation of invasive structures that degrade the ECM (Block et al., 2008; Murphy and Courtneidge, 2011; Linder et al., 2011). To determine whether p85β regulates cell invasion, we first studied its subcellular distribution and compared it with that of the other ubiquitous p85 subunit, p85α, which is generally expressed at higher levels in normal cells (Ueki et al., 2002). For p85α immunofluorescence (IF), we used an anti-SH3-p85α antibody (Ab); for p85β, we generated a polyclonal Ab (K1123) that does not recognize p85α (supplementary material Fig. S1).

**Fig. 1.**
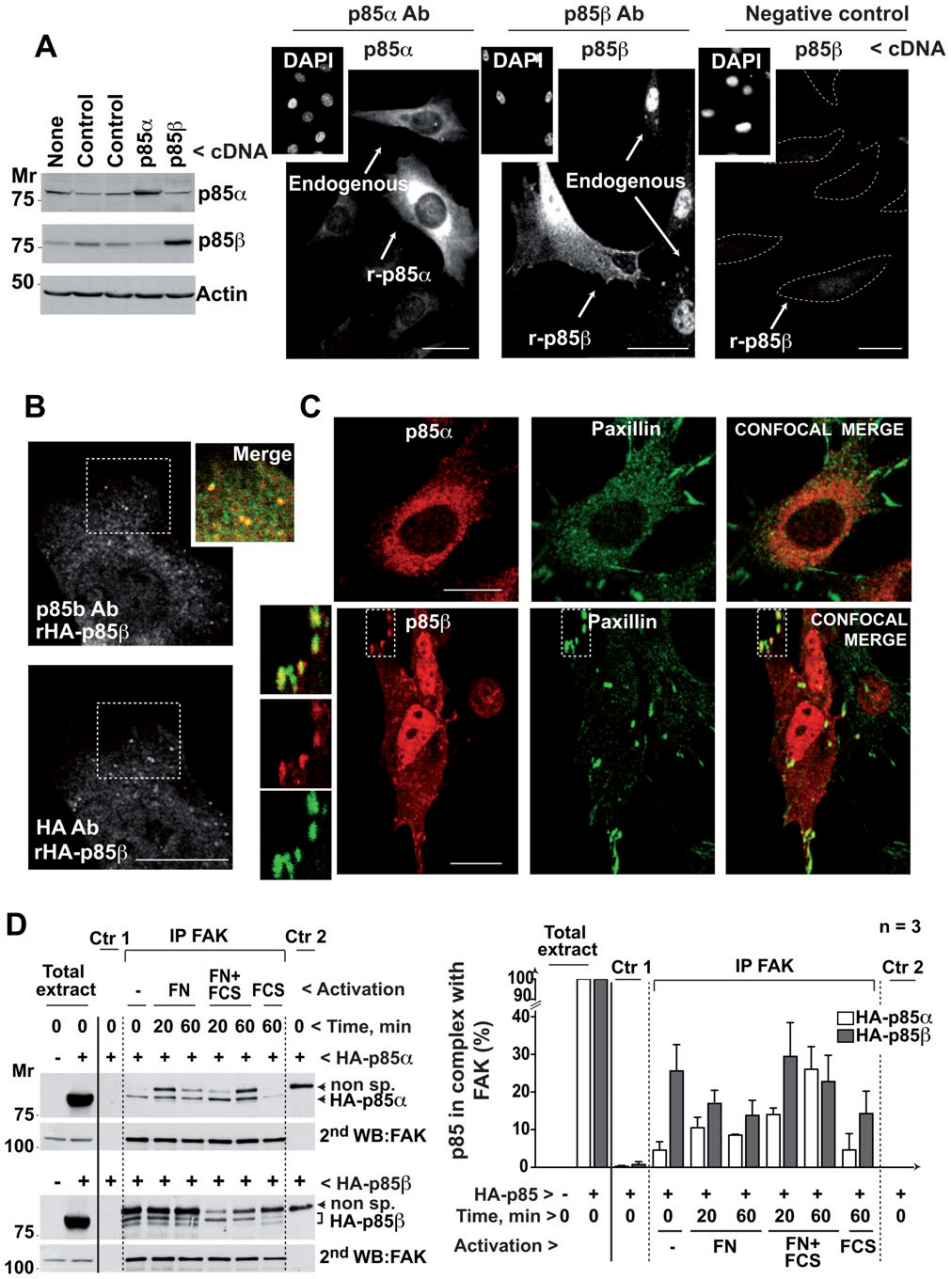
p85β localizes at adhesion plaques and associates with FAK. (A) NIH3T3 cells, untransfected or transfected with cDNA encoding p85α or p85β (48 h), were tested in Western blot (WB). Other cells were examined by immunofluorescence (IF) using p85α- or p85β (K1123)-specific antibodies. Insets show DAPI staining for nuclear DNA. To control K1123 specificity, some of the cells that were stained with the antibody (Ab), were preincubated with antigenic peptide. A dashed line outlines the cell membrane; transfected cells are indicated. (B) NIH3T3 cells transfected with HA-p85β cDNA were stained simultaneously with K1123 polyclonal Ab and an anti-hemagglutinin (HA) mAb; the inset shows a magnification of the indicated region. (C) NIH3T3 cells were transfected as above and tested in IF by simultaneous staining of paxillin and p85α or p85β. Insets show magnifications. (D) NIH3T3 cells were transfected with cDNA encoding HA-p85α or HA-p85β (48 h), then incubated without serum (16 h) and activated with 10% serum or in FN-coated wells with and without serum for 20 or 60 min. Cell extracts (500 µg) were immunoprecipitated with anti-FAK Ab. Negative controls included extract + protein A (Ctr 1) and protein A + Ab (Ctr 2); Extracts (50 µg) and immunoprecipitates were analyzed in WB using anti-HA Ab. Loading was controlled in WB using anti-FAK Ab. The graph shows HA signal intensity relative to HA-p85 expression in whole cell extract, considered 100% (mean ± s.d.; *n*  =  3). Bar  =  12 µm.

In NIH3T3 immortal fibroblasts, p85α concentrated in perinuclear intracellular membranes (Fig. 1A), as reported (Luo et al., 2005). Recombinant and endogenous p85α yielded a similar pattern, although the exogenous protein was expressed at higher levels (Fig. 1A). In contrast, p85β localized in the nucleus, as reported (Kumar et al., 2011), as well as in the first confocal z-section in contact with the matrix, where endogenous and recombinant p85β showed similar dotted staining patterns (Fig. 1A). Preincubation of cells with the p85β Ab plus its antigenic peptide eliminated most of the p85β signal, indicating Ab signal specificity (Fig. 1A). In hemagglutinin (HA)-p85β expressing cells, the dotted pattern identified by the anti-p85β Ab was similar to that observed using anti-HA Ab (Fig. 1B). The p85β Ab also stained adhesion-like structures in the BLM human metastatic melanoma cell line; staining was lost after siRNA reduction of p85β levels (supplementary material Fig. S2A).

To confirm the predominant p85β localization to cell adhesions, we simultaneously stained p85β (or p85α) and cell adhesion markers paxillin or vinculin (Block et al., 2008). p85β clearly concentrated in vinculin- and paxillin-positive adhesions, whereas p85α only stained a small proportion of these structures and concentrated in perinuclear internal membranes, including the cis-Golgi (Fig. 1C; supplementary material Fig. S2B,C) (Luo et al., 2005).

Given that the cells attach to the ECM through integrin receptors at cell adhesions (Gilcrease, 2007), we considered that p85β could associate with these receptors. Nonetheless, immunoprecipitation of β1 integrin receptors and western blot analysis of associated p85β did not reproducibly confirm this possibility (not shown). FAK is one of the first proteins recruited to focal adhesions (Mitra et al., 2005) and associates to p85 after cell adhesion (Chen and Guan, 1994; Hirsch et al., 2002). To compare p85α and p85β association with FAK using the same Ab, we used NIH3T3 cells that expressed similar amounts of recombinant HA-tagged p85α or p85β (rHAp85α or rHAp85β), at levels that were approximately double that of the endogenous proteins (not shown). Cells were activated with fibronectin (FN), with FN plus serum (FCS), or with FCS (in suspended cells). We immunoprecipitated FAK and tested for HAp85 association by performing a WB with anti-HA Ab. This blot revealed that rHAp85α and rHAp85β expression levels were similar, as well as a non-specific band of variable intensity that was present in control-2 lanes (Fig. 1D). The variable intensity of this band suggests that it is partially eliminated by the immuno-precipitation washes; we have not increased the stringency of the washing buffers to avoid impairment of FAK/HAp85 association. Nonetheless, under these conditions, the second blot performed with anti-FAK Ab demonstrated that the amount of FAK was highly similar in different conditions, excluding the possibility of distinct immunoprecipitation efficiency in different lanes. Despite the similarity in immunoprecipitated FAK in the different lanes, the amount of rHAp85β bound to FAK was five times higher than that of rHAp85α in quiescent adherent cells and in all activation conditions including serum addition in suspension; p85α only increased its association with FAK after cell activation with FN plus serum (Fig. 1D).

### p85β expression enhances cell adhesion in fibroblasts

**Fig. 2.**
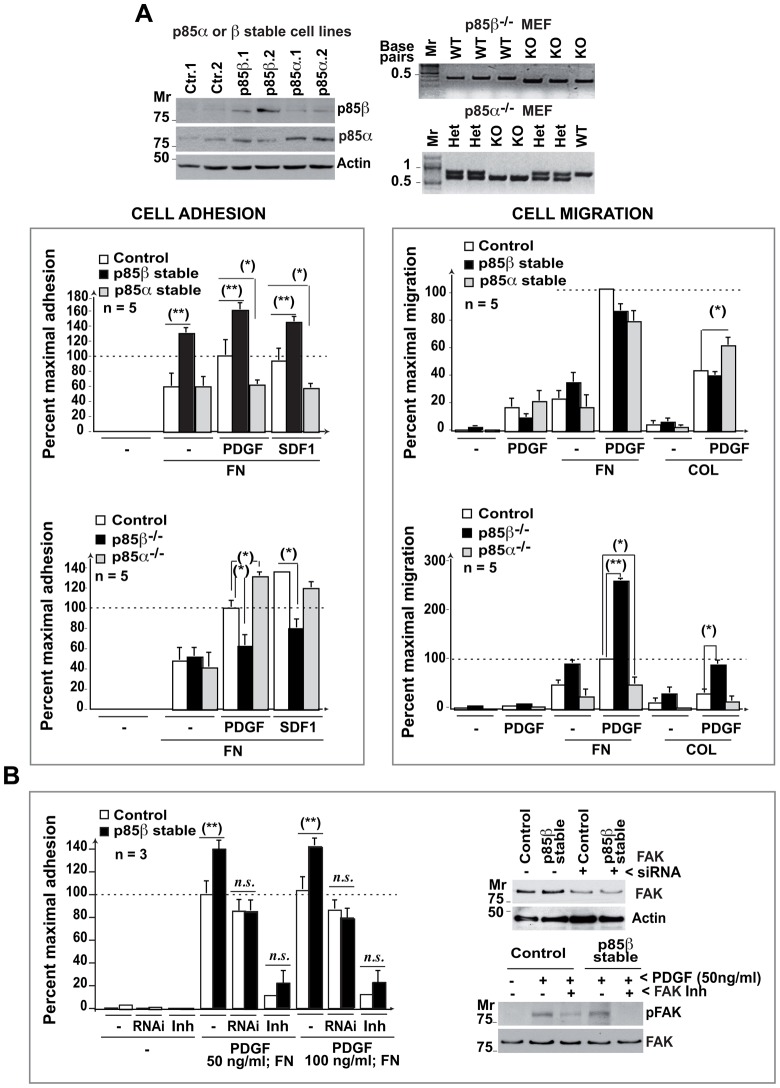
p85β regulates cell adhesion. (A) p85α or p85β expression was analyzed in WB in stably transfected NIH3T3 cells. Freshly isolated MEF (from WT, p85α^−/−^ or p85β^−/−^ mice) were genotyped by PCR. A fraction of the cells were activated with PDGF (50 ng/ml) and used in an adhesion assay, alone or on fibronectin (FN), or in a migration assay on transwells coated with a thin layer of FN or collagen (COL)(10 µg/ml). Graphs show the percent of response relative to the maximal in control cells (100%; mean ± s.d.; *n*  =  5). (B) Control and p85β-overexpressing NIH3T3 cells were transfected with FAK siRNA (48 h) or preincubated with FAK inhibitor (10 µM PF-573228) for 1 h at 37°C prior to stimulation with 50 or 100 ng/ml PDGF (indicated) on FN. The cells were assayed in an adhesion assay (as in A). Some of the cells transfected with siRNA or treated with the inhibitor were lysed and lysates examined in WB using were anti-FAK Ab or anti-pTyr576/577-FAK Ab (indicated). *n.s.* =  non-statistical significance; ** *P*<0.01, * *P*<0.05; Student's *t*-test.

Overexpression of p85α increases cell migration on collagen and reduces focal adhesion numbers and cell adhesion (Jiménez et al., 2000). To test whether p85β expression regulates cell adhesion or migration, we prepared and examined p85β-deficient MEF (murine embryonic fibroblasts) and p85β-overexpressing NIH3T3 cell lines (Fig. 2A). We confirmed that p85α overexpression reduces cell adhesion; in contrast, p85β overexpression increased and its deletion reduced cell adhesion to FN (Fig. 2A), showing that p85β expression enhances cell adhesion.

Increased cell adhesion induced by p85β expression might impair cell migration. Nonetheless, while p85β deletion enhanced platelet-derived growth factor (PDGF)-induced cell migration, p85β expression did not impair this process (Fig. 2A). We confirmed that p85α expression augmented cell migration on collagen and its deletion moderately reduced this process (Fig. 2A). Increased p85α levels thus promoted cell migration, while p85β expression triggered cell adhesion without impairing migration. Since p85β associates with FAK, we analyzed whether interference with FAK expression (using siRNA) or activity (using PF-573228 FAK inhibitor) impaired the cell adhesion advantage of p85β-overexpressing NIH3T3 cells. FAK siRNA transfection partially reduced FAK expression and FAK inhibition decreased the phosphoTyr576/577-FAK levels; both treatments eliminated the advantage on cell adhesion of p85β-overexpressing cells (Fig. 2B).

### p85β modulates cell adhesion structures

Formation of cell adhesions begins at the cell periphery in small focal contacts; these are transformed into elongated adhesion plaques that further mature into centrally localized fibrillar adhesions, which mediate strong adhesion (Block et al., 2008). To determine whether p85β action in adhesion was associated with an effect on cell adhesions maturation, we examined these structures in p85β-deficient or -overexpressing cells.

**Fig. 3.**
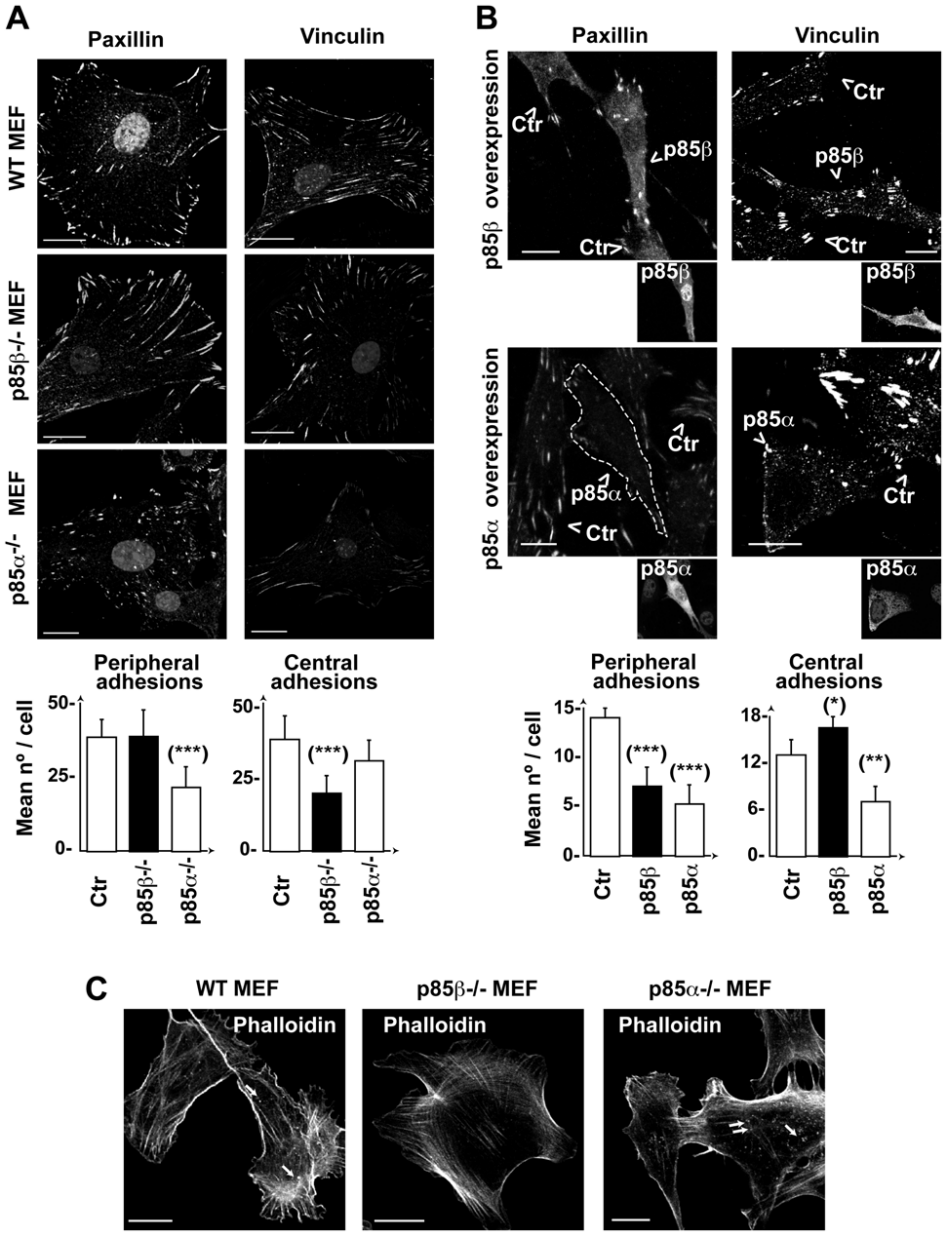
p85β modulates adhesion structures by increasing central adhesions. (A,B) WT, p85α^−/−^ or p85β^−/−^ MEF (A,C) or NIH3T3 cells transfected with p85α or p85β (B) were stained with anti-paxillin or -vinculin Ab. Images show the first confocal section from the adhesion plane. Transfected cells were identified by anti-p85β Ab (K1123) or -pan-p85 Ab (for p85α) (insets) and are indicated by an arrowhead. Graphs show the number of peripheral or central adhesions per cell (mean ± s.d.; *n*  =  25). Focal contacts and focal adhesions near the cell membrane were considered peripheral; rounded adhesions at the cell center (not in contact with the membrane) were considered central adhesions. (C) MEF as in (A) were stained using phalloidin-TRITC. Arrows indicate F-actin dots at the cell first z-section, in contact with the matrix. Bar  =  12 µm. *** *P*<0.001, ** *P*<0.01, *-*P*<0.05; Student's *t*-test.

Although p85α expression was necessary for the formation of peripheral focal contacts and adhesion plaques (that were markedly reduced by its deletion), p85α overexpression was also deleterious to these structures, as it reduced central and peripheral cell adhesions (Fig. 3A,B); this suggests that p85α expression influences both assembly and disassembly of cell adhesions. In contrast, p85β deletion selectively reduced centrally localized cell adhesions without disturbing peripheral contacts and adhesion plaques that were enlarged in these cells (Fig. 3A,B); this indicates that p85β is needed for development of peripheral into central cell adhesions. Accordingly, p85β overexpression increased the number of central adhesions and reduced peripheral focal contacts and adhesion plaques (Fig. 3B).

F-actin stress fibers attach to the plasma membrane at focal adhesions (Maruthamuthu et al., 2010). p85α overexpression reduces focal adhesions and stress fibers (Jiménez et al., 2000). We reasoned that p85α^−/−^ deletion would also decrease the stress fiber meshwork at the cell periphery as it reduces peripheral adhesions, whereas p85β deletion would not alter peripheral fibers, but would affect transcellular stress fibers anchored at the cell center. We confirmed these phenotypes (Fig. 3C), which supports a function for p85β in cell adhesions.

### p85β modulates F-actin polymerization at cell adhesions

**Fig. 4.**
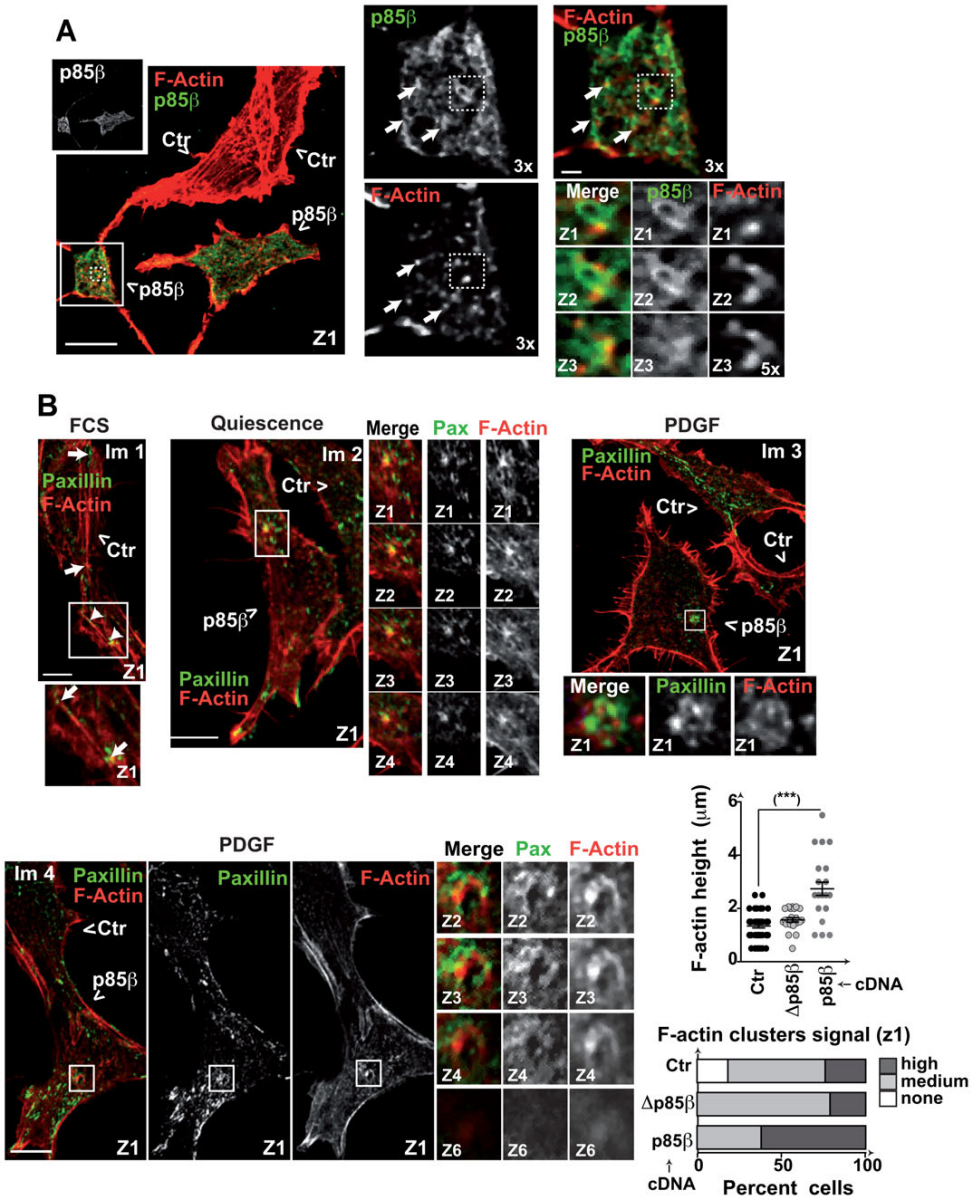
p85β expression induces formation of F-actin-positive adhesions. (A) NIH3T3 cells transfected with CFP combined with control or HA-p85β cDNA (48 h) were evaluated by immunofluorescence using anti-HA Ab and phalloidin-TRITC. Representative images of the first confocal section (z1). Insets show 3× magnification of indicated areas, small insets show 5× magnification of serial z-sections, arrows indicate areas of phalloidin and HA proximal staining. Transfected cells were tracked with CFP. (B) NIH3T3 cells were transfected with CFP combined with control HA-p85α, -p85β or -Δp85β cDNA (48 h). Cells were tested after incubation in serum-free medium (2 h), after activation with PDGF (50 ng/ml; 10 min) or in exponential growth (with serum). Cells were stained with phalloidin-TRITC and paxillin. The graphs show the percentage of paxillin^+^ clusters/rosettes with F-actin signals scored as high (similar to that of cortical F-actin), medium (lower than cortical F-actin signal), or none, and height of F actin^+^ clusters (µm) in different cells. Bar  =  12 µm. Step size, 0.5 µm. ***-*P*-<0.001; Student's *t*-test.

In MEF, phalloidin also stained F-actin-containing dotted structures at the cell base, which were fewer in p85β^−/−^ MEF (Fig. 3C). To determine whether p85β localized at these F-actin dots at the cell base, we transfected NIH3T3 cells with rHAp85β, and stained the cells with anti-HA Ab and TRITC (tetramethylrhodamine isothiocyanate)-labeled phalloidin. p85β localized in the vicinity of F-actin clusters at the cell base in serial z-sections (Fig. 4A, 5× magnification); some F-actin/p85β-positive dots formed groups larger than 10% the size of the nucleus (Fig. 4A, 3× magnification).

To determine whether the F-actin dots at the cell base indicated cell adhesions, we simultaneously stained F-actin and paxillin and tested the effect of p85β overexpression in these structures. We analyzed control and rHAp85β-expressing NIH3T3 cells in quiescence, in exponential growth (with serum), or after activation with PDGF-BB (50 ng/ml, 10 min), which triggers cell invasion (Rowe et al., 2009). We detected paxillin at the tips of the stress fibers (Fig. 4B, image 1), as reported (Maruthamuthu et al., 2010). We also found F-actin clusters that colocalized with the paxillin signal in several serial z-sections (Fig. 4B, image 2). These structures were detectable in distinct treatment conditions and cell types, but were more abundant in PDGF-treated p85β-expressing cells (0–1 clusters in controls, 2–4 in p85β-expressing cells) (Fig. 4B). Analysis of F-actin-positive cell adhesion clusters in z-sections showed greater cluster height and a higher F-actin signal in PDGF-treated p85β-expressing cells (Fig. 4B). The F-actin-positive cell adhesions detected in treated p85β-expressing cells resembled cell invasion structures (Murphy and Courtneidge, 2011).

To determine whether the p85β effect at increasing the height of F-actin/paxillin structures required PI3K activity, we analyzed cells expressing a p85β deletion mutant (rHAΔp85β) that does not bind to p110. Δp85β also localized near F-actin clusters at the cell base (supplementary material Fig. S3A); these cells showed a higher number of F-actin/paxillin adhesion clusters (1–3 per cell) and an increased F-actin signal in these structures compared to those of control cells in the first z-section, however, Δp85β expressing cells showed reduced z-axis height of F-actin/paxillin structures compared with those in p85β-expressing cells (Fig. 4B). p85β in complex with p110 thus induces F-actin polymerization in the z-axis.

### p85β is needed for the stability and height of BLM cell adhesions

**Fig. 5.**
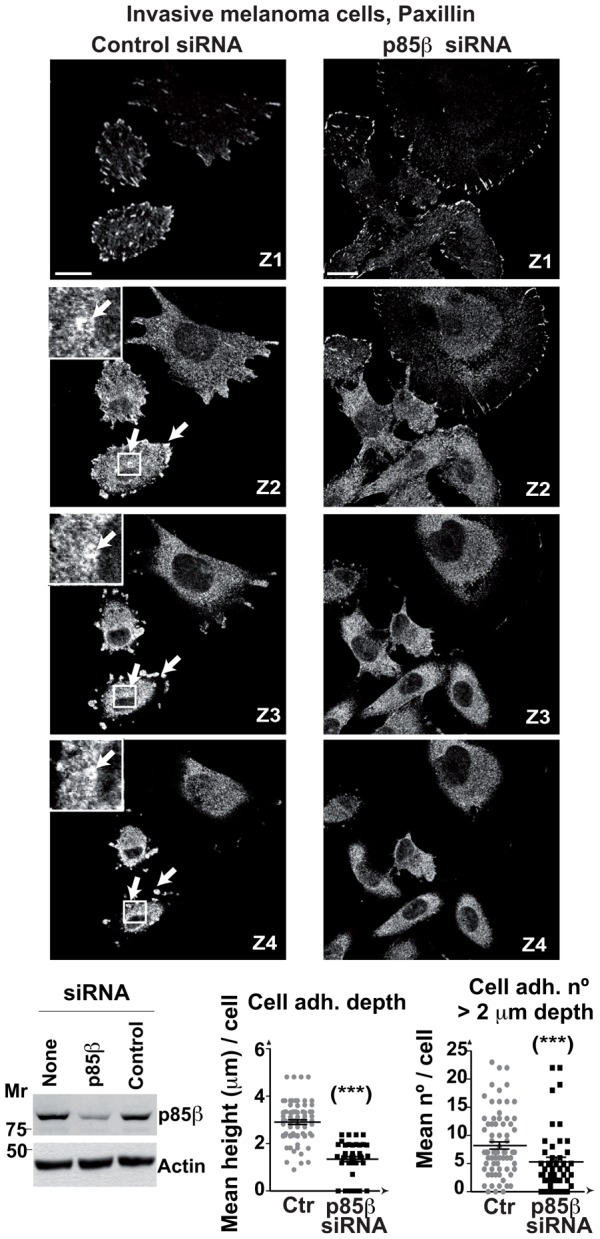
p85β is essential for maintenance of cell adhesions depth in melanoma cells. BLM cells were transfected with control or p85β siRNA (48 h), then stained with anti-paxillin Ab. Representative images at indicated z-sections; bar  =  12 µm. Arrows indicate adhesions that extended >2 µm in the z-axis (1 µm step size). Cell extracts were analyzed in WB. Graphs show the number of adhesions (>2 µm depth), and the mean depth of cell adhesions in control and p85β-depleted cells (mean ± s.d.; *n*  =  25). (***) *P* <0.001; Student's *t* test.

As p85β also concentrated at adhesion-like structures in melanoma BLM cells, we tested the consequences of depleting p85β in cell adhesions in these cells. Some BLM cells were spread onto the matrix and had numerous paxillin-positive adhesions at the cell adhesion plane; other cells had a smaller area in contact with the matrix (Fig. 5, z1). In the latter cells, adhesions showed paxillin signal in several serial z-sections (2 to 4 µm); some were organized in cluster/rosette-like structures (Fig. 5, inset). The most striking effect of p85β depletion in cell adhesions was height reduction although, as in fibroblasts, p85β depletion also induced accumulation of peripheral long adhesions and reduced the amount of centrally localized cell adhesions (Fig. 5, z1).

**Fig. 6.**
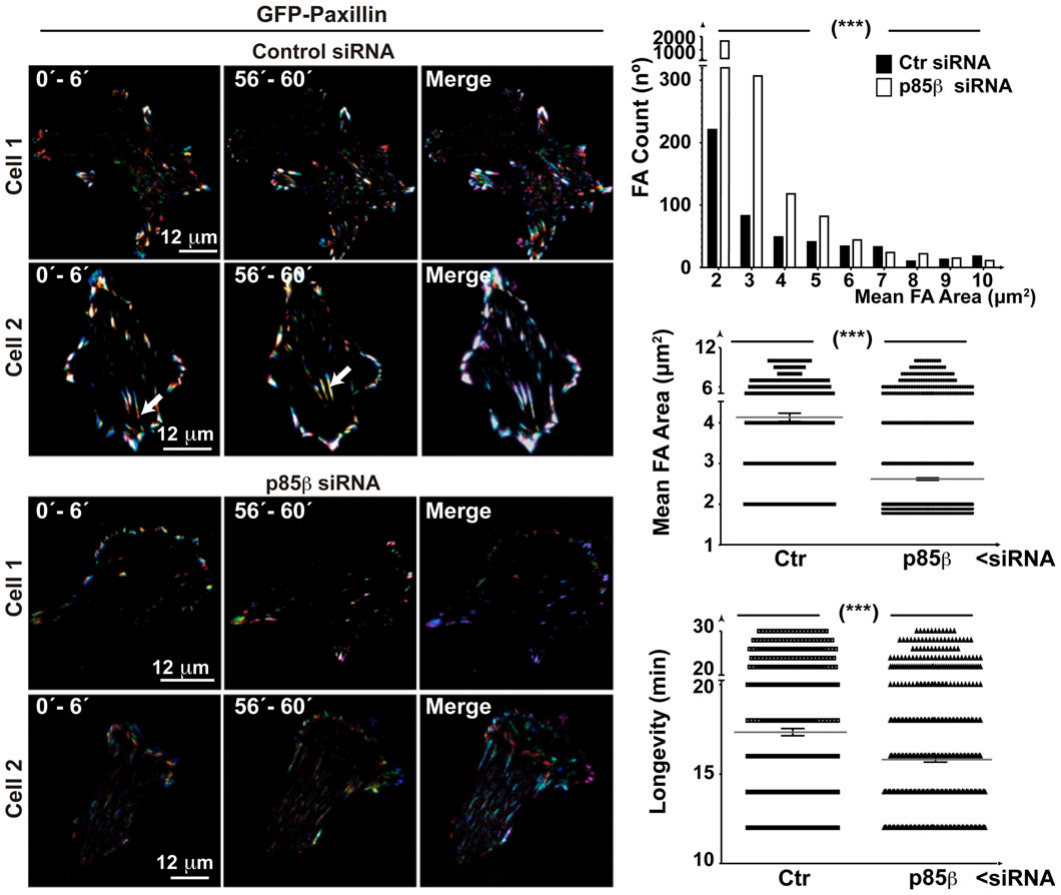
p85β mediates cell adhesion stability and maturation. BLM cells were transfected with control or p85β siRNA (24 h), then transfected with a plasmid encoding GFP-paxillin (24 h). TIRFM video microscopy was carried out at a depth of 70 nm. To evaluate the longevity and trajectory of cell adhesions, we assigned a different color to the paxillin signal collected in individual time frames and merged image sets; a white color (resulting from the mixture of colors) indicates long-lived adhesions. Left and center columns show the merge of three time points; the right column shows five time points spanning 30 frames (1 h). In control cells, some adhesions migrate to the cell center, with a parallel signal increase (indicated with an arrow). Graphs show the number of cell adhesions (top), differences in areas (center), and adhesion longevity (bottom) in *n*  =  12 cells in independent experiments. Mean ± s.e.m. are shown. *** *P* <0.001; Wilcoxon matched-pairs signed-rank test. Bar  =  12 µm.

The phenotypes induced by p85β expression in fibroblasts and BLM cells suggest that p85β induces maturation of peripheral contacts to central adhesions, as well as the development of F-actin-containing cell adhesions. We studied the p85β contribution to the dynamic evolution of cell adhesions using TIRFM (total internal reflection fluorescence microscopy), which permitted real-time analysis of cell adhesions adjacent to the attachment plane. BLM cells transfected with control or p85β siRNA in combination with green fluorescent protein (GFP)-paxillin were analyzed by TIRFM. To evaluate cell adhesion longevity and trajectory, we assigned a different color to the paxillin signal collected in individual time frames and merged image sets; a white color (resulting from the color mixture) indicated long-lived adhesions. Control cells showed a large number of stable adhesions, some of which moved towards the cell center (Fig. 6). In contrast, p85β depletion reduced paxillin intensity and area; adhesions seldom moved to the cell center and were significantly less stable (Fig. 6; supplementary material Movies 1, 2). These observations show that p85β increases cell adhesion size and stability, permitting maturation of small cell adhesions to larger, long-lived cell adhesions.

### p85β regulates invadopodium formation in melanoma cells

**Fig. 7.**
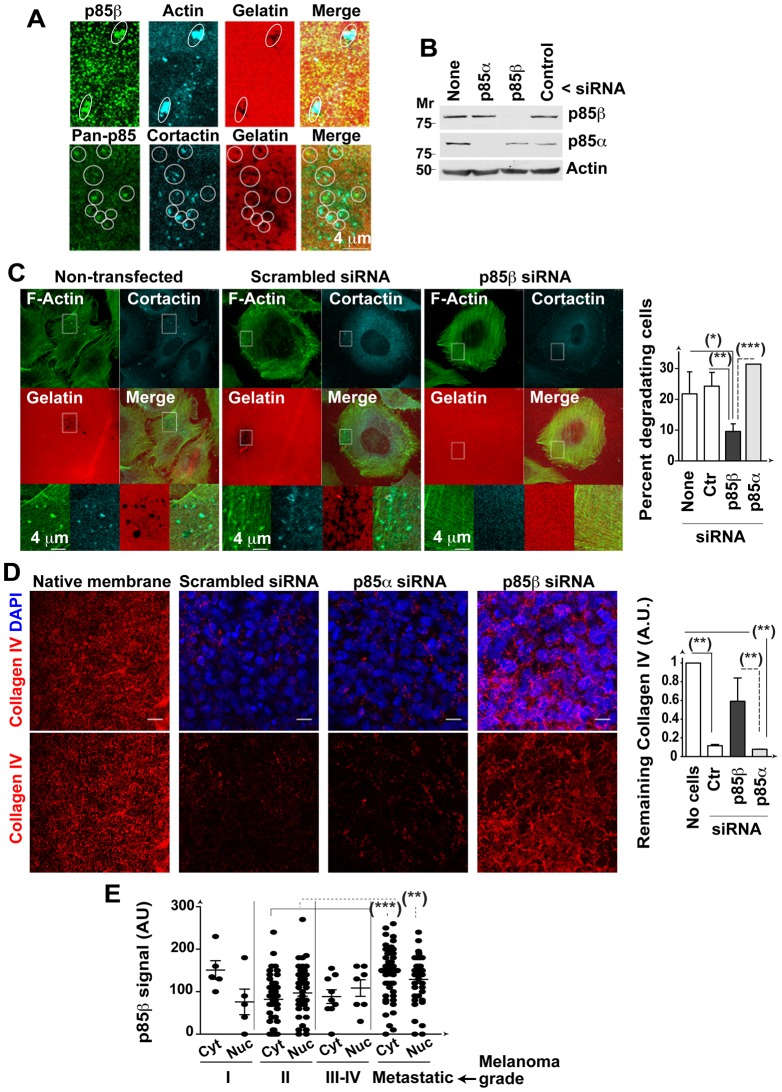
p85β is essential for melanoma invasion. (A) BLM cells in exponential growth were plated onto fluorescent gelatin-coated coverslips (6 h) and stained with anti-p85β or -pan-p85 Ab in combination with anti-cortactin Ab or phalloidin. White circles indicate areas in which p85 concentrates, which coincided with cortactin or actin clusters in >50% of cases. (B,C) BLM cells were transfected with p85α or p85β siRNA (48 h), plated onto coverslips and stained with phalloidin-FITC and anti-cortactin Ab. WB analysis confirms siRNA efficiency (B). Representative control or p85β silenced cells; insets show magnification of indicated areas. Graph shows the percentage of cells that degraded the matrix in distinct conditions (mean ± s.d.) (C). (D) BLM cells as in (C) were seeded on wild type mice basement membranes (72 h). Basement membrane invasion was analyzed by IF staining of collagen IV (red) and cell nuclei (DAPI, blue). The graph shows the signal (arbitrary units, AU) of the collagen signal remaining after incubation, determined in several z-sections (100%; mean ± s.e.m.; *n*  =  3). (E) p85β expression detected in immunohistochemistry using p85β Ab (K1123) in a tissue array of different grades of melanoma (indicated). The graph shows p85β signal intensity (AU) in the nucleus (Nuc) and cytosol (Cyt). Bar  =  4 µm. * *P* <0.05, ** *P* <0.01, *** *P* <0.001; Student's *t*-test.

Invadopodia form around an F-actin core (Albiges-Rizo et al., 2009). We hypothesized that p85β induced cell adhesion development into invadopodia by increasing F-actin deposition in the z-axis of cell adhesions. To determine whether p85β localizes to invadopodia and is necessary for their degradative activity, we cultured BLM cells on fluorescent gelatin and identified invadopodia by staining F-actin or cortactin (Murphy and Courtneidge, 2011). p85β staining with K1123 Ab or a pan-anti-p85 Ab (that mainly detects the p85β isoform at the cell base) showed that p85β concentrated at matrix degradation sites, where it codistributed with F-actin and cortactin (Fig. 7A). We also transfected BLM cells with control, p85α or p85β siRNA (48 h) and evaluated matrix degradation. Depletion of p85β, but not of p85α, reduced invadopodium formation and blocked gelatin degradation (Fig. 7B,C; supplementary material Fig. S3B), suggesting that p85β is needed for invadopodium formation in BLM melanoma cells.

We compared of the effect of p85α, Δp85β and p85β expression on the invasive capacity of NIH3T3 cells; p85β-expressing cells exhibited an invasive capacity markedly higher than control, p85α- or Δp85β-expressing cells (supplementary material Fig. S3C).

To confirm p85β requirement for cell invasion in a more physiological setting, we isolated native peritoneal basement membrane from C57BL/6 mice and tested the ability of control, p85α- or p85β-depleted BLM cells to degrade the basement membrane. Compared to controls or p85α silencing, p85β depletion reduced BLM cell capacity to degrade the basement membrane (Fig. 7D), confirming the need for p85β expression for BLM melanoma invasion.

As p85β triggered invadopodium formation in the BLM melanoma cell line, it is possible that high p85β levels confer an invasion advantage on metastatic melanomas. Using the anti-p85β Ab (K1123) in immunohistochemistry, we studied p85β expression in a melanoma tissue array. In normal skin we appreciated a moderate staining in keratinocytes and in the epithelial cells of sweat gland; in the few melanocytes present in these samples (*n* = 8), the signal was either absent or very low in the cytoplasm; skin fibroblasts were also negative (not shown). p85β signal was low in grade I-to-IV melanomas, but increased significantly in metastatic melanomas (Fig. 7E), showing that invasive melanomas express high p85β levels.

### p85β modulates Cdc42 and Rac activation and their localization to adhesion structures

p85β induced maturation of cell adhesions and generation of paxillin-positive adhesions with polymerized actin in the z-axis. Invadopodia differ from cell adhesions in their need for stable binding by GTP-Cdc42 and -Rac to maintain their F-actin skeleton (Tsuboi, 2007; Block et al., 2008). Since p85 associates to Cdc42 and Rac (Zheng et al., 1994; Jiménez et al., 2000), we tested whether p85β controlled the activity or localization of these GTPases.

**Fig. 8.**
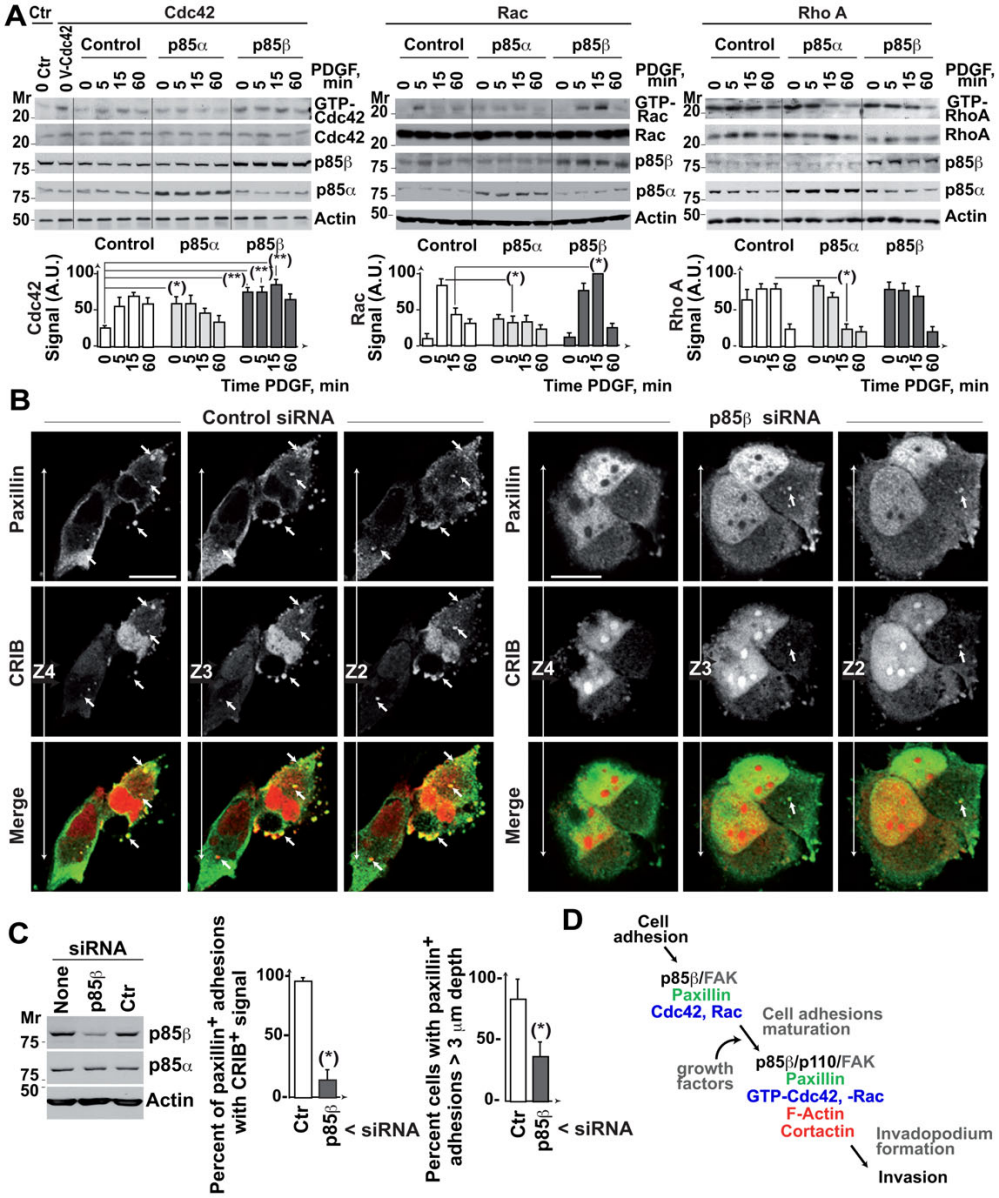
p85β regulates active Cdc42 and Rac localization to deep adhesions. (A) NIH3T3 cell lines expressing p85α or p85β were incubated without serum (2 h), then activated with PDGF (50 ng/ml) for the times indicated. A subset of cells was transfected with Myc-Cdc42. Cell extracts were incubated with purified bacterially bait (GST-rhotekin-RBD for RhoA, GST-Pak1 for Rac/Cdc42, GST-NWASP for Cdc42). As a positive control, NIH3T3 cells were transfected with V12-Cdc42. Total GTPase levels and pulled-down GTP-forms were analyzed in WB (using anti-Myc Ab for Cdc42). The graphs show normalized signal intensity (mean ± s.d.; *n*  =  3) (AU). (B) BLM cells were transfected with control or p85β siRNA in combination with CFP-CRIB^Pak1^ (48 h). The figure shows a representative image in three sequential z-sections. Arrowheads indicate cell adhesions that colocalize with active CFP-CRIB, some organized as rosettes. Bar  =  12 µm. (C) Reduction of p85β levels by siRNA transfection as analyzed in WB. Graphs show the percentage of paxillin positive adhesions containing CRIB signal and the percentage of cells showing adhesions of >3 µm depth. * *P* <0.05 ** *P* <0.01; Student's *t*-test. (D) When tumors augment p85β expression, cell adhesions increase their p85β/p110 content (in complex with FAK). This p85β increase, in the presence of growth factors, enables the local accumulation of GTP-Cdc42/Rac at cell adhesion and the generation of a z-axis F-actin core, necessary for invadopodium formation.

We examined p85α- or p85β-expressing cells. p85α expression increased basal GTP-Cdc42 and diminished PDGF-induced GTP-Rac and GTP-RhoA levels, as tested in pull-down assays (Fig. 8A). In contrast, p85β increased basal and PDGF-induced GTP-Cdc42 levels, increased PDGF-induced GTP-Rac levels, and did not reduce GTP-RhoA levels (Fig. 8A), showing that p85β enhances Cdc42 and Rac activation.

To test whether p85β mediates Cdc42 and Rac localization at cell adhesions, we expressed the cyan fluorescent protein (CFP)-fused CRIB^NWASP^ (that binds GTP-Cdc42) or CRIB^Pak1^ domain (that binds GTP-Cdc42/Rac) and tested their localization in p85α- or p85β-expressing cells. Nuclear CRIB localization was non-specific, as its size allows free nuclear entry. CRIB^NWASP^ and CRIB^Pak1^ localized to the plasma membrane and adhesion-like structures in control cells; p85α overexpression increased the plasma membrane CRIB signal, whereas p85β increased the CRIB signal in adhesion-like structures at the first confocal section (supplementary material Fig. S4A).

We also analyzed the effect of depleting p85β expression on GTP-Cdc42/Rac localization in cell adhesions in BLM cells. In control BLM cells, CRIB co-localized with paxillin in several serial z-sections; some CRIB-positive adhesions formed clusters/rosettes in the cell periphery and cell central region (Fig. 8B). In contrast, in p85β-depleted cells there was a smaller number of CRIB/paxillin-positive adhesions and, in the few detected, CRIB/paxillin colocalization extended for fewer sections than in controls (Fig. 8B,C). CRIB/vinculin staining yielded similar results (supplementary material Fig. S4B). p85β thus regulates GTP-Cdc42/Rac activity and localization at cell adhesions and is necessary for GTP-Cdc42/Rac localization in serial z-sections perpendicular to the cell matrix.

## DISCUSSION

The mechanism by which a tumor cell invades other tissues remains unclear. An increase in p85β expression parallels breast and colon carcinoma progression (Cortés et al., 2012). Here we analyzed p85β function in melanoma cell invasion. The presented results show that p85β in complex with FAK localizes at cell adhesions and mediates their stabilization, maturation, and z-axis actin polymerization. The mechanism of the p85β-mediated z-axis actin polymerization appears to involve GTP-Cdc42/Rac recruitment to cell adhesions, since p85β depletion reduced GTP-Cdc42/Rac localization to cell adhesion and their z-axis F-actin levels. This p85β function was PI3K activity-dependent, since p85β but not a mutant that does not bind p110, induced z-axis actin polymerization. p85β-dependent F-actin-rich adhesions behave as invadopodium precursors, since p85β depletion reduced these adhesions and blocked invadopodium formation and invasion. We show that clinical metastatic melanomas express high levels of p85β, suggesting that p85β favors invasion in melanoma. We propose that when tumors increase p85β expression, this results in p85β constitutive localization at cell adhesions (in complex with FAK), which in the presence of growth factors, enables accumulation of GTP-Cdc42/Rac at cell adhesions and generation of a z-axis F-actin core, necessary for invadopodium formation.

Analysis of the p85 subcellular distribution showed that, at difference from the isoform expressed at higher levels in normal cells, p85α, which accumulated in membranous structures and only associated with FAK in the presence of fibronectin and serum, p85β concentrated in cell adhesions and was bound constitutively to FAK. Indeed, FAK partial depletion or inhibition, eliminated the cell adhesion advantage of p85β-overexpressing cells, suggesting that FAK is necessary for the increase in cell adhesion mediated by p85β.

Whereas exogenous p85α overexpression reduced cell adhesion, p85β overexpression increased this process and induced maturation of focal/peripheral to round/central adhesions, some of which mediate strong adhesion (Block et al., 2008). Cell adhesions are distributed as foci on the ventral cell surface, concentrating integrin receptors and cytoskeleton contractile fibers that mediate cell attachment to the ECM (Wolfenson et al., 2013). Focal contacts are nascent cell adhesions that form behind the cell leading edge; these are transformed into longer structures (focal adhesions) that provide firm adhesion *via* actomyosin stress fibers; focal adhesions also develop into centrally located fibrillar adhesions that mediate strong adhesion (Wolfenson et al., 2013). The capacity of p85β to enlarge the number of centrally localized larger cell adhesions concurs with p85β enhancement of cell adhesion, as centrally localized adhesions mediate firm adhesion (Wolfenson et al., 2013). p85β involvement in cell adhesion maturation is also suggested by the TIRFM analysis, in this assay p85β depletion reduced paxillin intensity and area and impaired the movement of cell adhesions to the cell center; adhesions were significantly less stable (Fig. 6). These findings suggest that p85β increases cell adhesion size and stability, permitting maturation of small peripheral cell adhesions to larger, centrally localized long-lived cell adhesions. Initial adhesions develop into focal adhesions in a Rac1-dependent manner; assembly of mature focal and fibrillar adhesions requires RhoA (Heasman and Ridley, 2008; Block et al., 2008). Since p85β increased basal and PDGF-induced Cdc42 activation, enhanced Rac activation and, at difference from p85α, did not inhibit RhoA, the mechanism by which p85β induces cell adhesions maturation likely involves its action on the activity of Rho-GTPases.

p85β also induced formation of adhesions containing F-actin in the z-axis, resembling the invadopodium skeleton. Invadopodia and adhesion plaques share some protein components, but differ in their dependence on actin nucleation. Invadopodia are dynamic membrane protrusions associated with an active actin polymerization rate, whereas focal adhesions do not appear to nucleate actin (Yamaguchi et al., 2005; Block et al., 2008; Albiges-Rizo et al., 2009; Yu et al., 2012). To maintain their active F-actin skeleton, invadopodia must stably bind Cdc42/Rac (Tsuboi, 2007; Block et al., 2008; Etienne-Manneville and Hall, 2002). p85β mediated GTP-Cdc42/Rac localization at cell adhesions indicate a mechanism for Cdc42/Rac localization at these sites. Since Cdc42/Rac are needed for invadosome formation, p85β expression might trigger invadopodium formation by recruiting GTP-Cdc42/Rac to cell adhesions. Indeed, in melanoma cells, p85β depletion reduced the z-length of cell adhesions, their GTP-Cdc42/Rac levels, and cell invasion in collagen and in native basement membrane. In fibroblasts, p85β overexpression increased F-actin z-length at cell adhesions and cell invasion in collagen. These observations suggest that p85β regulates invasion by priming formation of invadopodium-like adhesion structures via a mechanism involving GTP-Cdc42/Rac recruitment to cell adhesions, which mediate actin polymerization.

The described action of p85β is consistent with invadopodium development from cell adhesions. This possibility is reinforced by the observation that invasive cancer cells have few focal adhesions and form invadopodia, whereas noninvasive cancer cells tend to form many focal adhesions and do not have invadopodia (Raz and Geiger, 1982; Oikawa et al., 2008). Some authors question invadopodium development from cell adhesions, as they found that inhibition of metalloproteinases in fibrosarcoma and mammary carcinoma cells induces a change from integrin-dependent elongated migration to amoeboid migration, which allows cells to invade by squeezing through the ECM, with little dependence on integrin receptors and proteases (Wolf et al., 2003; Sabeh et al., 2009). It is possible that although integrin-mediated adhesion might not be necessary in certain conditions, normally tumor cells interact with the surrounding ECM through integrin receptors and need proteases to degrade the matrix (Chang and Werb, 2001). Deficient integrin or protease activation could lead to selection for tumor cells with alternative modes of invasion; this is the case for melanoma cells migrate in a Rac-dependent elongated manner, but can adapt to Rho-dependent amoeboid migration (Sanz-Moreno et al., 2008; Pinner and Sahai, 2008).

The intracellular signals that trigger invadopodium formation are only partially known; Src and protein kinase C induce invadopodium formation, Pyk2 and cCbl regulate podosome assembly in osteoclasts, and FAK, Cdc42 and Rac GTPases also promote podosome and invadopodium formation (Hauck et al., 2002; Kaverina et al., 2003; Horne et al., 2005; Heasman and Ridley, 2008; Sanz-Moreno and Marshall, 2010). PI3K activity is important for invadopodium formation in head and neck carcinoma cells (Hoshino et al., 2012). According to our observations, p85β would form part of a central pathway induced at cell adhesions and involved in promoting invasion, which includes FAK, p85β/p110, GTP-Cdc42/Rac and F-actin. The extracellular signals that control cell invasion is also known only in part; integrin-dependent elongated migration appears to be induced by Tyr kinase and integrin receptors (Wolf et al., 2003; Sanz-Moreno and Marshall, 2010). Although p85β localized to cell adhesions independently of PI3K activity that is activated by extracellular signals, generation of F-actin-positive adhesions required associated PI3K activity; additionally, despite p85β expression increased basal GTP-Cdc42 levels, optimal Cdc42/Rac activation was only detected in p85β-expressing cells after PDGF treatment. Growth factors and ECM in the tumor microenvironment might trigger p85β-associated PI3K activity, which would synergize with the p85β scaffold function to induce GTPase activation and invadopodium assembly.

We show that low-grade melanomas have significantly lower p85β levels than metastatic melanoma, supporting a p85β function in clinical melanoma invasion. Low-grade melanoma has a partial “epithelial” gene expression pattern, but metastatic melanoma turns on again an invasive epithelial-mesenchymal transition genetic program (Gupta et al., 2005; Hoek et al., 2008 Kim et al., 2013; Caramel et al., 2013). Accordingly, whereas “in situ” melanomas express E-cadherin, metastatic “invasive” melanomas lack E-cadherin expression (Hoek et al., 2008; Kim et al., 2013; Caramel et al., 2013). *PIK3R2* (p85β) would represent one of the genes controlling cell invasion in advanced melanoma. Metastatic melanomas often express an active form of PIK3CA (p110α) (Kim et al., 2013; Hoek et al., 2008); in these cases, p85β upregulation could promote invasion even with limited supply of growth factors. The near-absence of F-actin/cortactin-positive clusters, GTP-Cdc42/Rac-containing adhesions, and matrix degradation in p85β-depleted melanoma cells, supports the idea that p85β localization might be a primary event in invadopodium formation in melanoma.

The presented results help to explain the acquisition of an invasive phenotype in advanced stages of colon, breast cancer (Cortés et al., 2012) and melanoma (Fig. 7), when these tumors increase p85β expression. p85β/p110 would localize at cell adhesions in complex with FAK, and, in the presence of growth factors, would enable accumulation of GTP-Cdc42/Rac at cell adhesions and generation of a z-axis F-actin core, necessary for invadopodium formation (schematic pathway in Fig. 8D).

## Supplementary Material

Supplementary Material
