# Supplementary material for: Phosphoinositide 3-kinase p85beta regulates invadopodium formation
Source: Biol Open. 2014 Sep 12;3(10):924–36. doi: 10.1242/bio.20148185 (PMC4197441; doi:10.1242/bio.20148185)
Supplement: Supplementary Material [file supp_3_10_924__index.html]

Phosphoinositide 3-kinase p85beta regulates invadopodium formation — Supplementary Material 

# Phosphoinositide 3-kinase p85beta regulates invadopodium formation

## bio.20148185 Supplementary Material

**Files in this Data Supplement:**

- Supplementary Material - Ariel E. Cariaga-Martínez et al. doi: 10.1242/bio.20148185
- Movie 1 - **TIRFM videomicroscopy of GFP-paxillin in control BLM cells.**
- Movie 2 - **TIRFM videomicroscopy of GFP-paxillin in BLM cells transfected with p85β siRNA.**
