# Supplementary material for: Phosphoinositide 3-kinase p85beta regulates invadopodium formation
Source: Biol Open. 2014 Sep 12;3(10):924–36. doi: 10.1242/bio.20148185 (PMC4197441; doi:10.1242/bio.20148185)
Supplement: Supplementary Material [file supp_bio.20148185_bio.20148185-s1.pdf]

Biology Open (2014) 000, 1–13 doi:10.1242/bio.20148185

Supplementary Material

Ariel E. Cariaga-Martínez et al. doi: 10.1242/bio.20148185

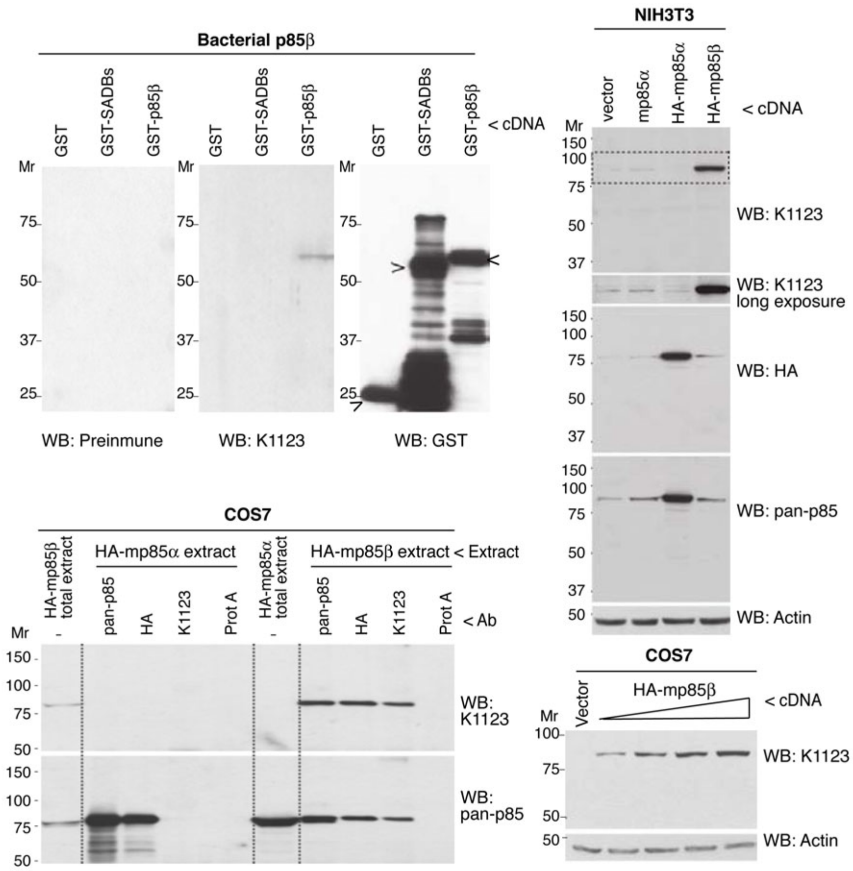

Fig. S1. The figure shows that K1123 antibody selectively recognized p85β but not p85α in immunoprecipitation (IP) and WB. The antibody was tested using bacterially produced GST fused to an N-terminal fragment of murine p85β or to an unrelated protein (GST-SADB). K1123 was also used for WB of total extracts of COS7 or NIH3T3 cells transfected with HA-p85α or -p85β. Increasing amounts of HA-p85β cDNA in COS7 cells augmented the p85β signal in WB. Transfected COS7 cells were also tested by immunoprecipitation and WB.

Biology Open (2014) 000, 1–13 doi:10.1242/bio.20148185

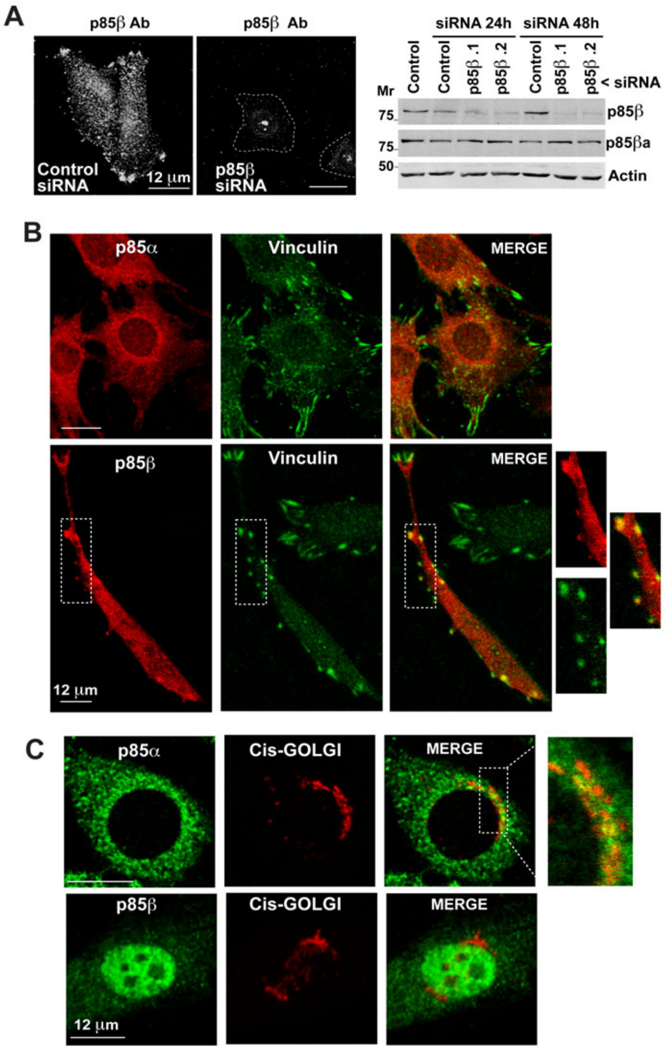

Fig. S2. (A) shows anti-p85β K1123 IF staining of BLM cells transfected with control or p85β siRNA, and the WB showing silencing efficiency. (B,C) NIH3T3 cells transfected with p85α or p85β and stained for vinculin and p85α or p85β (B), or simultaneous staining with a cis-Golgi marker and anti-p85α or -p85β (K1123) antibodies (C).

Biology Open

Biology Open (2014) 000, 1–13 doi:10.1242/bio.20148185

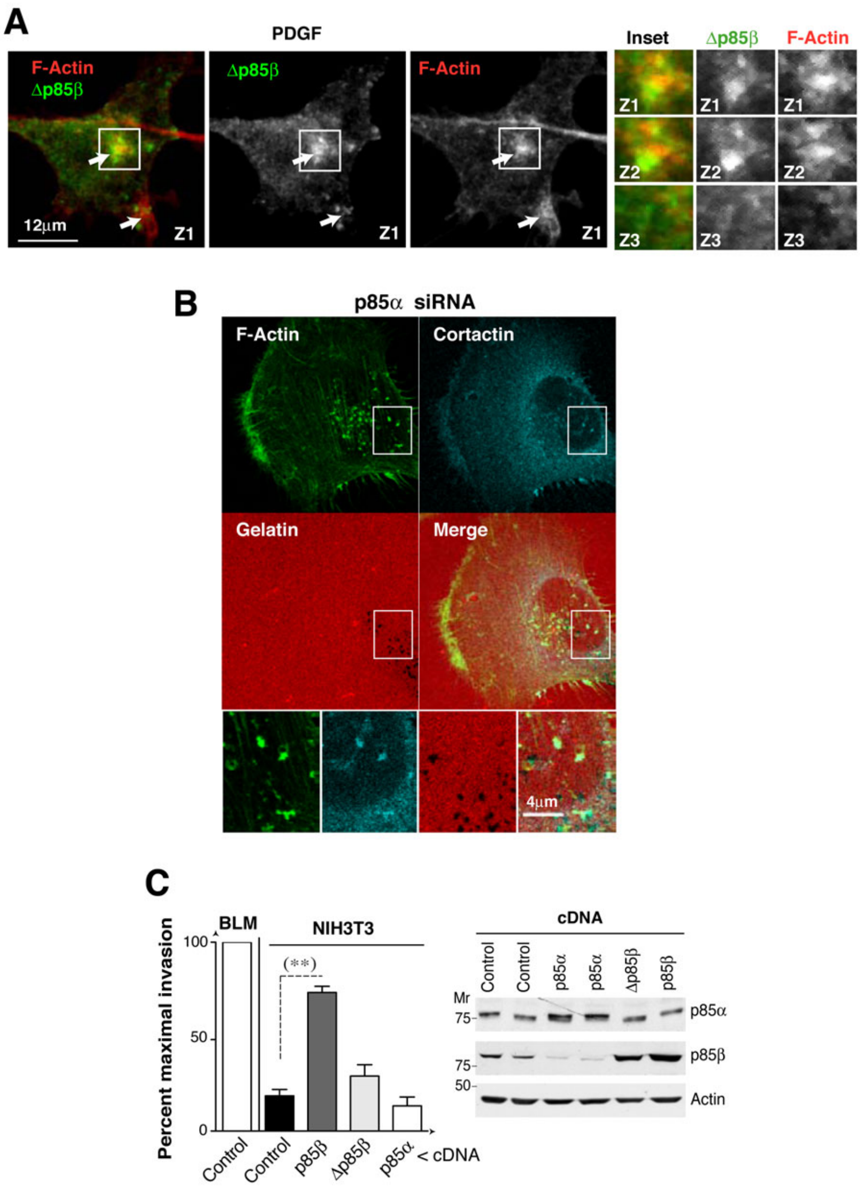

Fig. S3. (A) shows the simultaneous staining of F-actin and paxillin in cells transfected with HA-Δp85β and activated with PDGF. Insets show higher amplifications of z-sections. (B) Invasion analysis of BLM cells transfected with control or p85α siRNA (as in Fig. 7). (C) percentage of NIH3T3 cells transfected with indicated cDNA (48 h) that invade the matrix relative to maximum (BLM cells, 100%); protein expression was analyzed in WB.

Biology Open

Biology Open (2014) 000, 1–13 doi:10.1242/bio.20148185

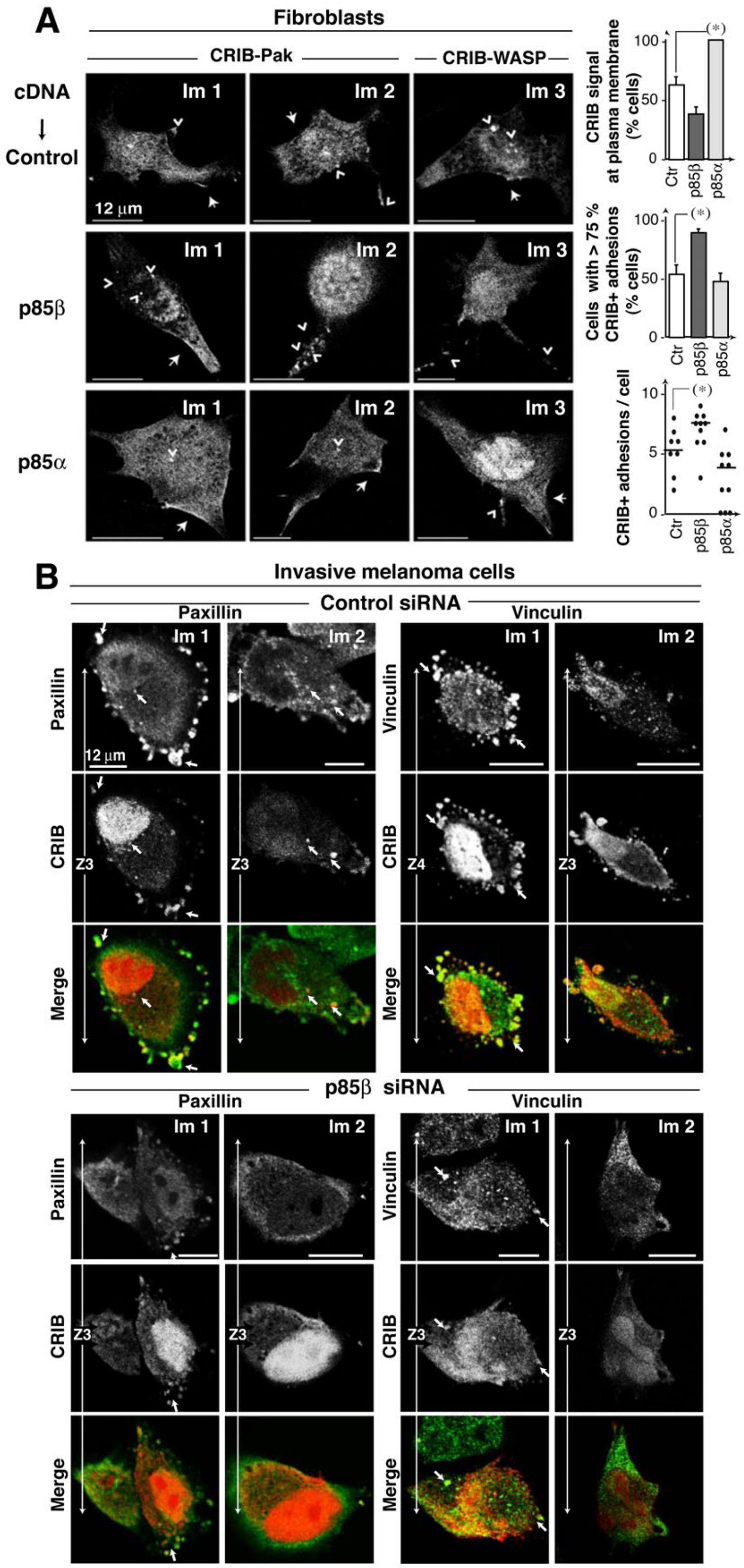

Fig. S4. (A) GTP-Cdc42 and -Rac localization in NIH3T3 cells expressing p85α or p85β, tested using CFP-CRIBPak1 or -CRIBNWASP; the graph shows the percentage of cells with indicated phenotypes and the number of CRIB-positive adhesion structures per cell. Arrows indicate plasma membrane; arrowheads, cell adhesions. Mean ± s.d.; $n = 25$. * $P<0.05$, Student's t test. (B) CFP-CRIBPak1 localization in vinculin-positive cell adhesions in control or p85β-depleted BLM cells.

Biology Open (2014) 000, 1–13 doi:10.1242/bio.20148185

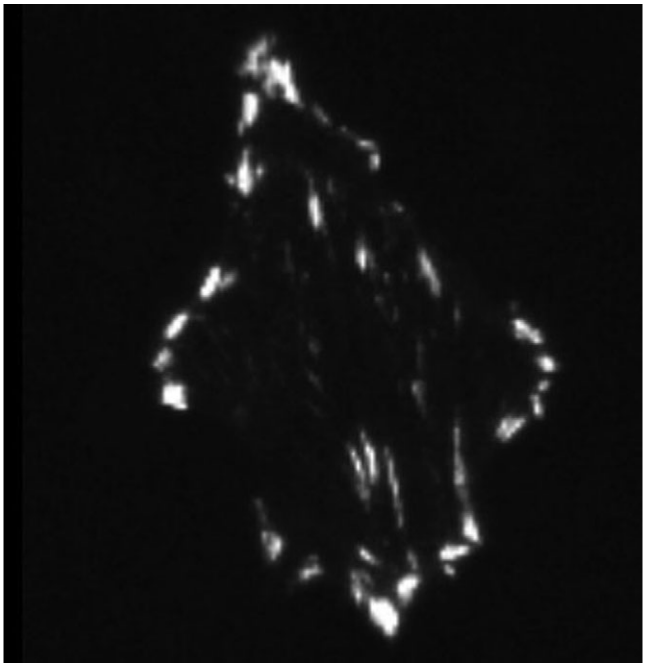

Movie 1. TIRFM videomicroscopy of GFP-paxillin in control BLM cells.

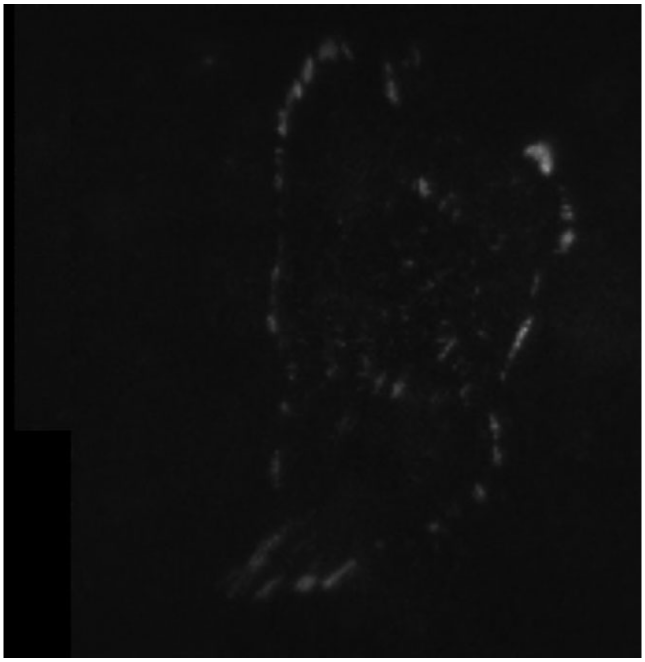

Movie 2. TIRFM videomicroscopy of GFP-paxillin in BLM cells transfected with p85β siRNA.
